# PKR Activation Favors Infectious Pancreatic Necrosis Virus Replication in Infected Cells

**DOI:** 10.3390/v8060173

**Published:** 2016-06-21

**Authors:** Amr A.A. Gamil, Cheng Xu, Stephen Mutoloki, Øystein Evensen

**Affiliations:** Faculty of Veterinary Medicine and Biosciences, Norwegian University of Life Sciences, P.O. Box 8146 Dep., 0033 Oslo, Norway; amr.gamil@nmbu.no (A.A.A.G.); cheng.xu@nmbu.no (C.X.); stephen.mutoloki@nmbu.no (S.M.)

**Keywords:** PKR, cloning, PKR inhibitor, IPNV, eIF2α phosphorylation, necrosis

## Abstract

The double-stranded RNA-activated protein kinase R (PKR) is a Type I interferon (IFN) stimulated gene that has important biological and immunological functions. In viral infections, in general, PKR inhibits or promotes viral replication, but PKR-IPNV interaction has not been previously studied. We investigated the involvement of PKR during infectious pancreatic necrosis virus (IPNV) infection using a custom-made rabbit antiserum and the PKR inhibitor C16. Reactivity of the antiserum to PKR in CHSE-214 cells was confirmed after IFNα treatment giving an increased protein level. IPNV infection alone did not give increased PKR levels by Western blot, while pre-treatment with PKR inhibitor before IPNV infection gave decreased eukaryotic initiation factor 2-alpha (eIF2α) phosphorylation. This suggests that PKR, despite not being upregulated, is involved in eIF2α phosphorylation during IPNV infection. PKR inhibitor pre-treatment resulted in decreased virus titers, extra- and intracellularly, concomitant with reduction of cells with compromised membranes in IPNV-permissive cell lines. These findings suggest that IPNV uses PKR activation to promote virus replication in infected cells.

## 1. Introduction

Type I interferon (IFNα/β) responses constitute some of the crucial innate responses against virus infections. The induction of these responses begins with the stimulation of IFN production that can be induced through different pathways [1]. The produced IFN binds its receptor on the cell surface and induces signaling cascades through the JAk/STAT pathway. As a result, an antiviral state is created characterized by increased transcription of several antiviral IFN stimulated genes (ISGs). Examples of these genes are those encoding the myxovirus resistance protein (MX), Protein kinase R (PKR) and ISG15 [2].

PKR is one of the best-studied ISGs and has been shown to play important roles during development, stress responses and virus infections. Genes encoding PKR have been characterized in different mammalian and fish species [3,4]. Analysis of amino acid sequences of PKR from different species revealed a common structure consisting of amino-terminal regulatory domain containing the dsRNA binding motif (dsRBM) and carboxy-terminal catalytic domain containing 11 kinase subdomains [4,5]. Phylogenetic analysis and amino acid sequence alignment of the kinase domain from mammalian and fish PKR revealed low sequence conservation although some of the domains that are important for dimerization or substrate interaction were conserved [3].

The classical mode of action of PKR is the induction of translation arrest in order to prevent production of new virus progeny thereby limiting virus spread. For this to happen, PKR must first be activated by binding of the dsRNA and other specific type of RNAs to its binding motif [6,7,8]. Once bound, a series of phosphorylation events are induced that ultimately lead to PKR activation. Activated PKR phosphorylates the eukaryotic initiation factor α (eIF2α), the only known substrate so far [9]. Phosphorylation of eIF2α prevents the recycling of GDP to GTP by the guanine nucleotide exchange factor eIF2B, which is important for the function of the translation initiator ternary complex Met.tRNA-eIF2-GTP, resulting in global inhibition of protein synthesis [10].

Recently, PKR was found to be involved in apoptosis, formation of stress granules (SGs) [11,12] and IFN-induced cellular necrosis [13]. Induction of apoptosis by PKR can be initiated via the death receptor, caspase 8 mediated or mitochondrial, caspase 9 mediated pathways; and involves both NFkB activation and eIF2α phosphorylation [11,14]. IFN-induced necrosis, on the other hand, requires IFN as well as interaction between PKR and RIP1 (receptor-interacting protein 1) to trigger necrosome formation and is licensed by FADD and caspases [13]. The detailed mechanism by which PKR participates in forming the SGs is not well described although it has been shown to involve eIF2α phosphorylation [15].

Infectious pancreatic necrosis (IPN) is an important disease of farmed Atlantic salmon (*Salmo salar* L.). The causative agent, IPN virus (IPNV), is a non-enveloped virus and is classified under the family *Birnaviridae*, genus *Aquabirnavirus*. The virus is ~60 nm in diameter and consists of a capsid that contains two genome segments (A and B) of double-stranded RNA [16]. The relatively small segment B encodes the virus polymerase, VP1 [17] whereas segment A contains a large open reading frame (ORF) encoding a 107-kDa polyprotein (NH_2_-pVP2-VP4-VP3-COOH). This polyprotein is post translationally cleaved to produce the major structural proteins VP2 and VP3 by the action of viral protease (VP4) [18,19,20]. A second ORF overlapping the N-terminal region of VP2 encodes a small non-structural protein VP5 [21,22].

At cellular level, IPNV-infected cells exhibit morphological and functional signs of necrosis [23] but the events preceding the necrotic stage are less known. Some IPNV strains are reported to induce necrosis in infected cells in a post-apoptotic phenomenon [24] while others induce necrosis without being preceded by cellular apoptotic events or only to a very limited extent [25,26]. In a previous study, we showed that IPNV infection of permissive cells resulted in eIF2α phosphorylation but PKR expression was not increased. Infected cells showed membrane leakage and reduced viability at 48 h post infection, but this could not be linked to PKR involvement [25]. Here, we have cloned Atlantic salmon PKR, developed custom made antibodies against it and investigated its role during IPNV infection with the aim to understand the involvement of PKR in virus replication and release of virus from infected cells. We did not observe any increase in PKR protein expression post IPNV infection but despite so, inhibition of PKR using the small molecule inhibitor of PKR C16 resulted in reduced eIF2α phosphorylation. Moreover, release of virus to the supernatant was reduced by 2 log_10_ in two permissive cell lines upon treatment with C16. A similar reduction was observed for the intracellular viral load. IPNV infected and C16 treated cells exhibited a lower number of cells with compromised membranes compared to untreated-IPNV infected cells. These findings indicate that PKR plays a role in promoting IPNV replication in infected cells.

## 2. Materials and Methods

### 2.1. Cell Lines

Chinook salmon embryonic (CHSE-214) cells [27], TO cells [28] and Asian Grouper strain K (AGK) cells [29] were maintained in L-15 media with Glutamax^®^ (Gibco, Carlsbad, CA, USA) supplemented with 5%–10% FBS (Sigma Aldrich, St. Louis, MO, USA). For maintenance, CHSE-214 and TO cells were grown at 20 °C while AGK cells were kept at 28 °C. *Epithelioma papulosum cyprini* (EPC) cells were maintained at 20 °C with L-15 medium with Glutamax^®^ (Gibco) supplemented with 5% fetal bovine serum (FBS, FBS), l-glutamine, and gentamicin.

### 2.2. Virus Propagation

A virulent recombinant IPN virus (rNVI-15R^b^), carrying threonine at positions 217 and 247, and alanine at position 221 of VP2, previously produced by reverse genetics [30] was used. For propagation, the virus was inoculated into 70%–80% confluent AGK cells and incubated at 15 °C until full CPE. The supernatant containing the virus was then harvested and clarified by centrifugation at 2500 rpm, 4 °C for 10 min. The concentration of the virus was estimated by titration in 96 well plates (Falcon, Bedford, MA, USA) containing 90%–100% confluent CHSE-214 cells.

### 2.3. Cloning, Prokaryotic Expression of Salmon PKR and Production of Rabbit Antiserum

Total RNA from TO cells that had been treated with recombinant IFNα as previously described [31] was used as a template for cDNA synthesis. Transcriptor first-strand cDNA synthesis kit (Roche, Basel, Switzerland) was used to make cDNA according to the manufacturer’s instructions. For initial cloning, one pair of primers, PKR-F1 and PKR-R1, was designed on the basis of Atlantic salmon PKR mRNA sequence (GenBank accession No. EF523422). A region from 73 bp upstream of the start codon of the open reading frame (ORF) to 412 bp downstream of the stop codon was amplified. The PCR products were purified by using the QIAquick gel extraction kit (Qiagen, Hilden, Germany) and cloned into the pGEM-T Easy vector (Promega, Madison, WI, USA). The ORF of salmon PKR gene was subcloned from pGEM-T into the prokaryotic vector pET-32c (Novagen, Madison, WI) by using primer set pET32c-PKR-F and pET32c-PKR-R. The recombinant vector containing a 6× His-tag at the N-terminal of the protein was used to facilitate purification using a His-Bind column. The recombinant vector, named pET32c-PKR, was confirmed by DNA sequencing and transformed into the bacterial host BL21 (DE3) for expression driven by the T7 polymerase. Induction was carried out at 37 °C for 2 h with 1 mM Isopropyl-β-d-Thiogalactopyranoside (IPTG). The fusion protein was purified according to the protocol of the His-Bind purification kit (Novagen) and used to immunize a rabbit for production of polyclonal anti-PKR serum. Immunization was done at the University of Life Sciences, Laboratory of Experimental Animals, and according to national legislation for the use of experimental animals.

### 2.4. Recombinant IFNα Treatment

To test the effect of IFNα treatment on the expression of salmonid PKR, CHSE-214 cells grown in 6 wells plates (Corning Life Science, Lowell, CA, USA) were treated with 500 ng/mL IFNα as described previously [31] and harvested at 4, 8, 16, 24 and 48 h post treatment. Parallel wells were left untreated and harvested together with cells treated for 48 h. At the indicated times post treatment, cells were sampled for Western blot and real-time PCR.

### 2.5. Effect of IPNV Infection on PKR Expression

Six well plates containing approximately 90% confluent CHSE-214 cells were infected sequentially in reverse order with 20 PFU/cell IPNV to produce cells infected for 3, 12, and 24 h at the time of sampling. Negative and positive controls were uninfected cells and cells treated with recombinant IFNα (500 ng/mL of medium), respectively, harvested after 4 days. The cells were sampled by washing once with PBS prior to lysis and Western blot analysis.

### 2.6. Western Blot

Following IPNV infection or recombinant IFNα treatment, CHSE-214 cells were lysed by using CelLytic M reagent and scraped from the plates. Lysates were separated in 12% NuPAGE Bis-Tris gels (Invitrogen) and transferred to PVDF membrane using Trans-Blot SD semi-dry transfer cell (BioRad, Hercules, CA, USA). The membrane was blocked for 2 h using 5% dry milk in TBST (0.02 M Tris-HCl, 0.9% NaCl, 0.05% Tween 20, pH 7.6) and then incubated overnight at 4 °C with polyclonal antibodies against PKR diluted in 5% dry milk in TBST. Horseradish peroxidase (HRP) conjugated anti-rabbit antibody (GE healthcare, Piscataway, NJ, USA) diluted 1:2000 was added and incubated for 1 h. The signal was finally developed using SuperSignal West Dura Extended Duration Substrate (Pierce, Rockford, IL, USA) or the ECL Plus^TM^ Western Blotting detection reagents and detected using the Typhoon (GE healthcare). After detection, the membrane was washed twice with 0.1% TBST, stripped using Restore^TM^ Plus buffer (Pierce), washed twice as above, and re-probed using antibodies against actin (Sigma Aldrich) as described above.

### 2.7. Quantitative Real-Time PCR Analysis

To quantify the expression of PKR following IFNα treatment RT-qPCR was used. Cells were treated with IFNα, as described above, and total RNA was isolated by using the RNeasy Plus minikit (Qiagen) according the manufacturer’s instructions. RNA concentrations were determined by using the Nanodrop ND1000 (NanoDrop technologies, Wilmington, DE, USA). One microgram of total RNA was used to synthesize cDNA using a Transcriptor first strand cDNA synthesis kit (Roche) according to the manufacturer’s protocol. The cDNA was diluted 1:5 and stored at −20 °C until required.

Quantitative PCR was performed in 96 well plates using the LightCycler 480 system (Roche). For each reaction, 2 µL cDNA was mixed with 10 pmol gene-specific primers and 10 µL LightCycler 480 SYBR green I master mix (Roche). The final concentration was adjusted to 20 µL using RNase free water. The sequences of primers used in the reactions are provided in Table 1. The cycling conditions for the PCR reactions were as follows: denaturation at 94 °C for 10 s; annealing at 60 °C for 20 s; elongation at 72 °C for 8 s. The results were analyzed by the ∆∆CT relative quantification approach [32] using β-actin as a reference gene.

### 2.8. Pre-Treatment of Cells with PKR Inhibitor C16 on eIF2α Phosphorylation

Both PKR inhibitor C16 and its inactive form (Merck Millipore, Billerica, MA, USA) were dissolved in DMSO to 5 mg/mL. CHSE-214 cells were then incubated with 2 µM of either solution for 30–45 min at 15 °C. Parallel wells of pretreated and untreated cells were subsequently infected with IPNV for 24 h, as described above, in the presence or absence of C16. Uninfected cells, both from C16 treated and untreated cells, were also included as controls. At 24 h post infection (hpi), cells were lysed and lysates were purified using ReadyPrep TM 2-D cleanup kit (BioRad). Thirty micrograms of purified lysates were subjected to 2D-Western blot analysis to evaluate the levels of phosphorylated eIF2α (peIF2α) in infected and uninfected cells. Samples were first subjected to isoelectric focusing (IEF) using 11 cm, nonlinear, pH 3–10 IPG strips (BioRad) followed by second dimension run using 8%–16% Tris-HCl Criterion precast gels and finally blotting into PVDF membrane before being probed using monoclonal anti human eIF2α antibodies (Cell Signaling, Beverly, MA, USA). Anti-mouse IgG HRP labeled secondary antibodies (GE healthcare) were applied and the signal was developed using SuperSignal West Dura Extended Duration Substrate (Pierce) and finally detected using the Typhoon (GE healthcare).

### 2.9. Flow Cytometry

CHSE-214 or TO cells grown in 24 wells plates were either pre-treated for 30 min with 2 µM C16 or left untreated. Three wells of each (C16 pretreated and untreated cells) were then infected with IPNV at 20 PFU/cell, and incubated for 24 h in the presence or absence of the PKR inhibitor, respectively. Uninfected cells in triplicate were also included as negative controls. After 24 h incubation, the media was removed and cells were washed once with PBS before trypsinization. Trypsinized cells were re-suspended in FBS and centrifuged for 10 min at 300× *g*. Cells were then stained using Annexin V/Ethidium homodimer III (EtD-III) staining kit (Biotium, Hayward, CA, USA) according to the manufacturer’s protocol, in order to determine the fraction of cells with apoptotic (Annexin V positive) or membrane permeability (EthD-III positive) changes. Flowcytometry was performed for 10,000 events with a Guava easyCyte^TM^ Flow Cytometer (Merck Millipore) and the acquired data were analyzed using InCyte^TM^ software version 0.2 (Merck Millipore). The following parameters were measured: (1) the area pulse of forward light scatter (FSC-A) *vs.* side scatter (SSC-A); and (2) fluorescent intensities of CF^TM^ 488A (filter 525/30) and EthD-III (filter 690/50) upon excitation with 20 mW 488 nm laser. Cell aggregates and debris were identified and excluded by using the width pulse of FSC-A *vs.* area width of SSC-A.

### 2.10. Effect of PKR Inhibition on Virus Loads in the Supernatant

To determine the impact of PKR inhibition on IPN replication, confluent CHSE-214 or TO cells seeded in 24 wells plates (Corning) were either pretreated for 30–45 min with either an active or inactive form of the PKR inhibitor C16 (Merck Millipore) as described above, or left untreated. Cells were then infected with IPNV at 20 PFU/cell and after one hour adsorption, they were washed once with PBS. After washing, L-15 glutamax media (Invitrogen) supplemented with 1% FBS and 50 µg/mL Gentamycin was added to the cells. For cells pre-treated with either forms of the PKR inhibitor C16, 2 µM of the inhibitor was also included in the media. Supernatants were harvested at 10, 24 and 48 hpi and the amounts of virus in the supernatants were determined by titration in CHSE-214 cells using Kärber’s method [33].

### 2.11. Effect of PKR Inhibition on the Intracellular Virus Loads

Intracellular virus loads were determined in cells infected with IPNV in the presence or absence of C16, as described above. At 10, 24 and 48 hpi, supernatants were removed from the cells. Attached cells were then washed once with PBS. Then, 200 µL of fresh cell culture media was subsequently added to the cell layers, which were then lysed by subjecting them to two rounds of freeze-thawing in order to release virus to the supernatant. The amount of virus released from the cells was finally determined by titration in CHSE-214 cells as already described.

### 2.12. Effect of Over-Expressing a Catalytic, Inactive Form of PKR

Eukaryotic expression plasmid encoding a mutated version of carp PKR (pcDNA-mutcarpPKR, Lys419Arg), which expresses a catalytically inactivated form, was a kind gift from professor Gui [34]. For over-expression of carp PKR proteins, EPC were transfected by electroporation with 2 µg of pcDNA-mutcarpPKR or pcDNA3.1-myc-His per 106 cells using the Neon transfection system (Invitrogen) with one pulses of 1200 V for 40 ms. After transfection, cells were kept at 20 °C until further examination.

### 2.13. Statistics and Graphics

Differences in virus titers between groups were determined by Wilcoxon Rank Sum test using JMP 11 statistical software (SAS institute Inc., Cary, North Carolina, USA). Two-way analysis of variance (ANOVA) followed by a Bonferroni post-test was performed to compare the differences in the percentages of necrotic cells between the different groups. The ANOVA analysis and all the graphs were done using GraphPad Prism 5.0 (GraphPad Software Inc., San Diego, CA, USA).

## 3. Results

### 3.1. Production of Rabbit Antiserum against Atlantic Salmon PKR

**Figure 1:**
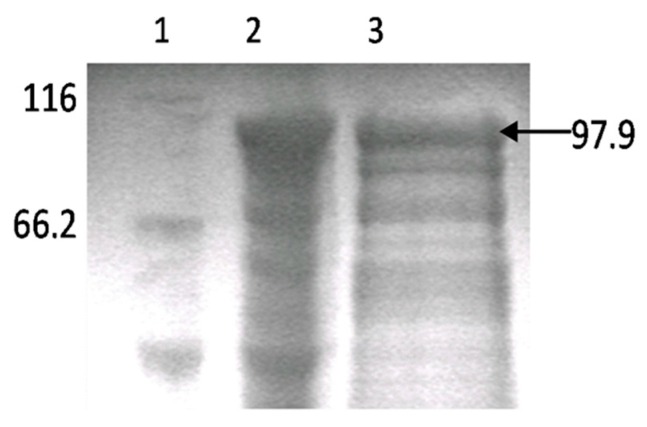
Expression of recombinant PKR protein in *E. coli* and its purification. 1. Protein marker; 2. pET32-PKR induced with IPTG; 3. Purified, recombinant PKR of a size of 97.9 kD.

Polyclonal antiserum against PKR (anti-PKR) was made by immunizing a rabbit with recombinant proteins produced by expressing the ORF of PKR in Escherichia coli BL21 (DE3) cells using the pET prokaryotic expression system. Purification of proteins was performed using His-Bind columns under denaturing conditions because the protein yield in the cell soluble fraction was much lower than that of the inclusion bodies. The expression and purification of recombinant proteins were identified by SDS-PAGE (Figure 1). The predicted molecular weight in CHSE-214 cells is around 85 kDa which corresponds to the size of trout PKR (Accession No. NM_001145891). The polyclonal antibody was used to detect PKR expression in CHSE-214 cells in response to recombinant IFNα treatment and IPNV infection.

### 3.2. PKR Expression in CHSE-214 Cells Is Induced following IFNα Treatment

**Figure 2:**
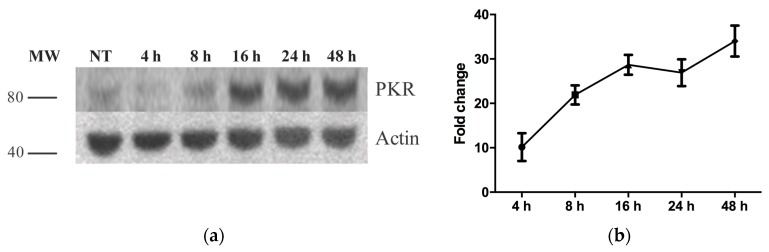
Time course analysis of PKR protein (**a**) and mRNA (**b**) expression in CHSE-214 after recombinant IFN-α treatment detected by Western blot using 1:1000 dilution of the custom-made antibodies (**a**) and real-time PCR (**b**). The real-time data are expressed as the mean fold changes in gene expression for IFN-α treated samples relative to the untreated control after normalization to β-actin (*n* = 2). MW = molecular weight; NT—non-treated; different time points are post IFN treatment.

To confirm that the antibodies described in the section above bind to PKR in CHSE cells using Western blot, the latter were pretreated with recombinant IFNα. Thereafter, anti-PKR antibodies were used to detect PKR expression. This approach was used since it is well-known that PKR expression is induced following IFNα pretreatment. The results show that while PKR is constitutively expressed at low levels in untreated cells, a strong induction was observed from 16 h post IFN-α treatment (Figure 2a). These findings correlate with PKR mRNA expression by real time PCR (Figure 2b).

### 3.3. PKR Expression Is Not Induced in IPNV Infected Cells

**Figure 3:**
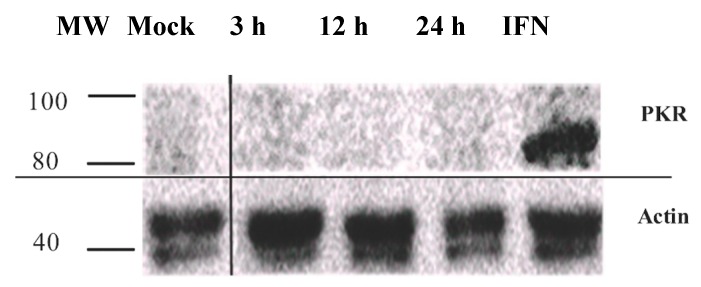
PKR expression in cells infected with infectious pancreatic necrosis virus assessed by Western blot analysis. The cells were harvested at indicated times (hours; h) following infection. IFN = cells were treated with interferon α for four days prior to sampling. MW = molecular weight.

PKR has previously been suggested to be involved in mediating cell death during IPNV infection [35]. To test this, the anti-PKR antibodies (above) were used. Our findings show that PKR is not induced in IPNV infected cells at any of the time points examined (3–24 hpi) while IFNα treated cells (positive controls) showed induction of PKR expression (Figure 3).

### 3.4. Pretreatment of Cells with PKR Inhibitor Alters eIF2α Phosphorylation after IPNV Infection

**Figure 4:**
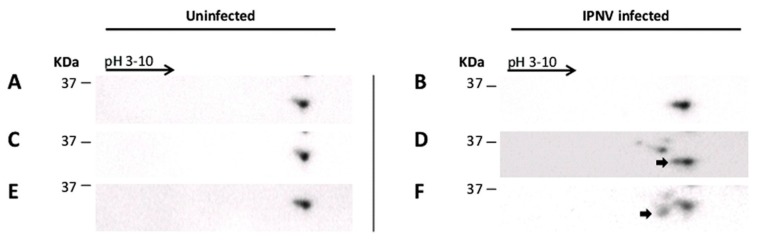
Effect of PKR inhibitor C16 on eIF2α phosphorylation (CHSE-214 cells). Parallel wells of C16-treated (**A**,**B**); C16 inactive form (**C**,**D**) or C16-untreated cells (**E**,**F**) were infected with 20 pfu/mL IPNV (**B**,**D**,**F**) or left uninfected (**A**,**C**,**E**). Non-infected cells pretreated with activated C16 (**A**), non-activated C16 (**C**) or left untreated (**E**) all exhibited an identical protein pattern. IPNV/pretreated with active C16 (**B**) showed inhibition of eIF2α phosphorylation. IPNV/pretreatment with inactive C16 (**D**) gave shift of eIF2α phosphorylation towards acidic pH (small tale; arrow in **D**). IPNV alone (**F**) gave increased eIF2α phosphorylation, shift to acidic pH (protein band corresponds to eIF2α (36 kDa). Total cell lysates fractioned on 2D gels and subjected to Western blot using anti-eIF2α antibodies. Arrows indicate the shift of eIF2α to acidic pH from phosphorylation.

Constitutively expressed basal levels of PKR can contribute to eIF2α phosphorylation when activated. Given that eIF2α was previously found to be phosphorylated following IPNV infection [25,36], we wanted to know whether PKR is involved in this phosphorylation. We therefore pretreated cells with the PKR inhibitor C16 followed by infection with IPNV and then observed eIF2α phosphorylation through the course of infection. Since salmonid antibodies against phosphorylated PKR are presently not available, PKR phosphorylation could not be measured directly, instead eIF2α phosphorylation was used as a downstream indicator. IPNV infection alone resulted in increased eIF2α phosphorylation as indicated by a shift in the protein band corresponding to the size of eIF2α (36 kDa) towards more acidic pH (Figure 4F). Pretreatment of cells with C16 followed by IPNV infection inhibited eIF2α phosphorylation (Figure 4B). Pretreatment of cells with inactive C16 also resulted in some decreased eIF2α phosphorylation (Figure 4D), presumably due to spontaneous activation. It is also noteworthy that in all lysates, non-specific bands (>40 kDa) were detected in addition to specific ones but no effort was directed towards identifying them. These findings suggest that PKR contributes to eIF2α phosphorylation in IPNV infected CHSE-214 cells.

### 3.5. Inhibition of PKR Reduces Virus Yield in the Supernatant

**Figure 5:**
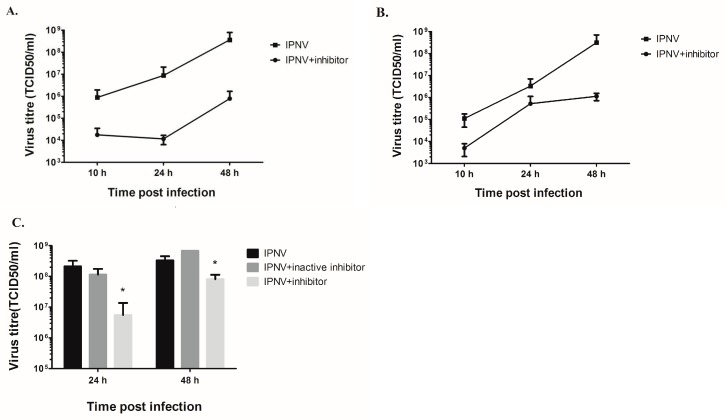
Effect of the PKR inhibitor C16 on IPNV replication. CHSE-214 (**A**) and TO (**B**) cells were either treated with 2 μM of the PKR inhibitor C16 or left untreated and then infected with IPNV at 20 PFU/mL and the amount of virus titers in the supernatants at different times post infection was determined by titration in CHSE-214 cells. Data represent an average of six parallels taken from two independent infections ± SD; (**C**) Virus titers in the supernatants in CHSE-214 cells following pretreatment with active and inactive form of C16 compared to untreated cells. Data represent an average of three parallels from a single infection ± SD. Asterisk indicate statistical significance compared to IPNV infected cells (*p* < 0.05).

To further investigate the role of PKR activation during IPNV infection we used two different cell lines to measure the effect of inhibitor on virus loads in the supernatants by titration. For CHSE-214 cells (Figure 5A), treatment with PKR inhibitor resulted in a significant reduction of virus titer in the cell supernatant by 1.7 log_10_ at 10 hpi (*p* = 0.0126), over 2.5 log_10_ by 24 (*p* = 0.0033) and 48 hpi (*p* = 0.0039). The same effect was seen in TO cells (Figure 5B) where the virus titer in the supernatant was reduced 1.3 log_10_ at 10 hpi (*p* = 0.0039), 0.8 log_10_ at 24 hpi (*p* = 0.037) and, thereafter, 2.4 log_10_ at 48 hpi (*p* = 0.0036) in C16-treated cells.

To validate that the effect observed on virus titer is due to PKR activation, we treated CHSE-214 cells with an active and inactive form of C16 and compared the virus loads on the supernatants to untreated cells at 24 and 48 hpi. We found that treatment with the inactive form of the inhibitor had a minor impact on virus titer (±0.3 log_10_ change) while treatment with the active form of C16 resulted in a significant reduction (*p* < 0.05) in virus titers by 1.5 log_10_ at 24 hpi and 0.6 log_10_ at 48 hpi (Figure 5C).

Since PKR knock-out cell lines of piscine origin are not available, we used an approach to transfect permissive cells (EPC cells) with a catalytic inactive form of PKR. EPC cells were transfected with pcDNA-mutcarpPKR, which will out-compete the function of cellular PKR [34]. Transfected cells were infected with IPNV at 1 and 0.1 MOI, which resulted in a significant reduction of endpoint titer at 1 MOI (not significant at 0.1 MOI; Supplementary Figure S1), further corroborating the importance of functional PKR for IPNV replication in permissive cell lines.

### 3.6. Inhibition of PKR Results in Decreased IPNV-Induced Membrane Damage

**Figure 6:**
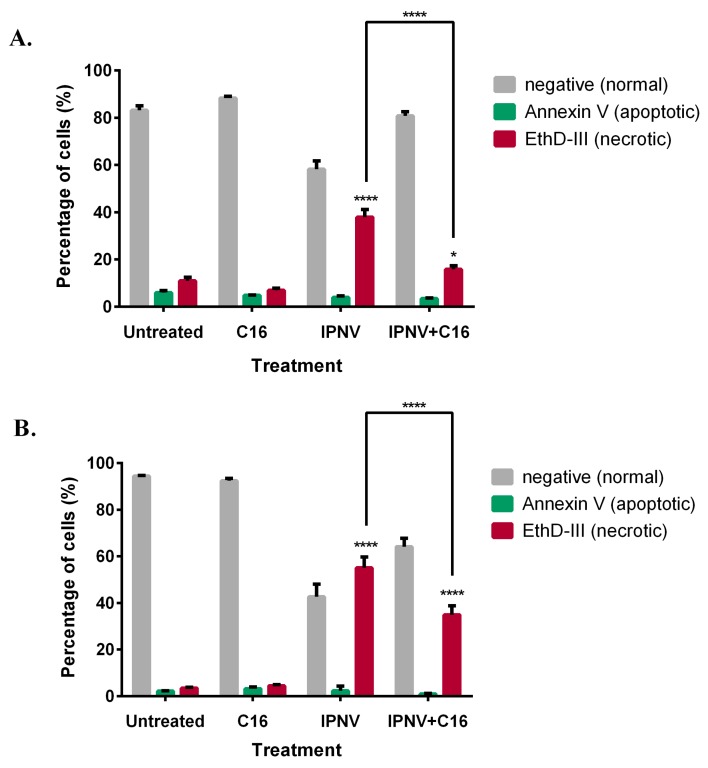
PKR induced membrane damage promotes virus release from infected cells. CHSE-214 (**A**) and TO (**B**) cells were infected with IPNV for 24 h in the presence or absence of the PKR inhibitor C16 and apoptotic (Annexin-V) or membrane permeability (EthD-III) changes were assessed by flow cytometry as described in the methodology. The number of cells with compromised membranes (EthD-III) was significantly reduced by C16 treatment while the number of apoptotic cells (Annexin-V) remained unchanged. Bars represent the mean of three parallels ± SD. Asterisks indicate statistical significance; * *p* < 0.05, **** *p* < 0.0001, *n* = 3.

To understand the mechanism behind the decreased virus titers, we further tested whether treatment with the PKR inhibiter would affect the ability of the virus to compromise the cell membrane, a well-known trait of IPNV [25]. We compared membrane permeability and apoptotic changes following IPNV infection in untreated and C16 treated cells. In both CHSE and TO cells, IPNV infection resulted in an increase in the percentages of cells with compromised membranes (EthD-II positive) compared to uninfected cells (Figure 6). No significant effect on the percentage of apoptotic cells (Annexin-V positive) was observed (Figure 6). Notably, however, the percentage of cells with compromised membrane was significantly lower in cells treated with C16. The decrease in the number of cells with compromised membranes was associated with the delay in CPE; with the CPE starting at 24 hpi in IPNV infected cells untreated or treated with inactive form of the C16 while evident CPE was only observed at 48 hpi in C16 treated cells.

### 3.7. The Decrease in Membrane Damage and Virus Replication Results from Inhibition of Virus Replication

**Figure 7:**
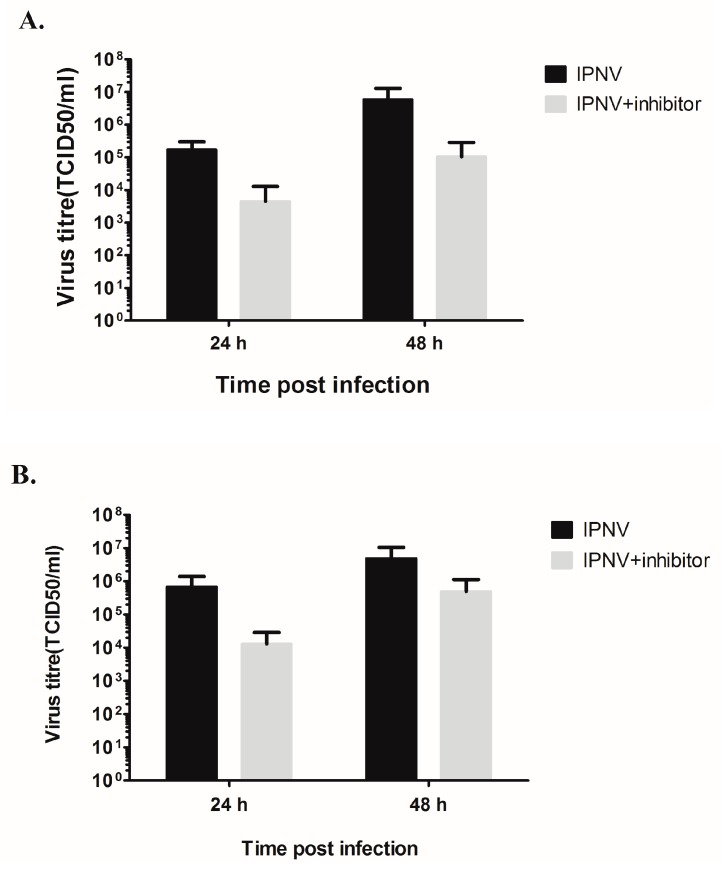
Effect of the PKR inhibitor C16 on the intracellular virus loads. CHSE-214 (**A**) and TO (**B**) cells were either treated with 2 μM of the PKR inhibitor C16 or left untreated and then infected with IPNV at 20 PFU/mL. The virus was released from attached cells by 2 rounds of freeze-thaw at different time post infection followed by titration in CHSE-214. Data represent an average of six parallels taken from two independent infections ± SD.

At this point, there are two explanation for the observed reduction in virus titer and the virus induced cell damage; either the PKR mediates the induced membrane damage which subsequently promotes virus release, and therefore inhibition of PKR blocks virus release or the effect is due to an effect on the virus replication. If the former is true then the virus will accumulate in the infected cells and a higher virus titer in the intracellular compartment can be expected. On the contrary, a lower virus titer is expected in C16 treated cells if there is a direct effect on IPNV replication. To clarify this point, the virus amounts intracellularly were assessed by titration of washed, freeze/thawed cells. In CHSE-214 cells (Figure 7A), there was a significant reduction in titer by 1.6 log_10_ at 24 hpi (*p* = 0.0038) and by 1.75 log_10_ at 48 h (*p* = 0.0038). The amount at 10 hpi was undetectable. Similar to CHSE-214, virus titer occurred intracellularly at 10 hpi below detection limit in TO cells, whereas there was a 1.7 (*p* = 0.0038) and 1 log_10_ (*p* = 0.0435) reduction in virus titers at 24 and 48 hpi, respectively (Figure 7B).

## 4. Discussion

In this study, we show that inhibition of PKR results in decreased virus titer in two IPNV permissive cells lines, strongly indicating that a functional PKR favors IPNV replication. The use of PKR activation in favor of the virus has been shown previously for other virus species. For example, hepatitis C virus (HCV) replicates more efficiently when PKR is activated and inhibition of PKR activation decreases virus yield [37]. This has been attributed to inhibition of IFNα production resulting from the global inhibition of protein synthesis induced through PKR activation. Our previous findings [25] suggest that the same IPNV strain used in this study uses inhibition of protein synthesis as a strategy to evade IFN responses. In the present study, we additionally show that inhibition of PKR activation results in decreased virus titer intracellularly and in the supernatant (Figure 5 and Figure 7). The level at which the PKR inhibitor interferes with virus replication is not clear but the decrease in intracellular titers suggests that it occurs prior to the viral release stage, possibly at replication levels. Interference at multiple stages is also a possibility. The detailed mechanism underlying this interference remains unknown and requires further investigation. It is known that PKR activation results in transcriptional activation of genes and several transcription factors involved in different cellular processes some of which could be beneficial for virus replication. For example, it was previously shown that activation of the transcription factor ATF3 (activating transcription factor 3), whose transcription can be induced by PKR [38], is beneficial for murine cytomegalovirus (MCMV) infection [39]. Similarly, the NFkB (Nuclear factor kappaB) is an important transcription factor that is activated by PKR and it has been shown that activation is to the advantage of several virus species [38,40]. One possibility could therefore be that activation of some of the PKR-induced genes or transcription factors promotes IPNV replication and inhibition of their activation consequently hampers virus replication but this remains to be decided.

Since PKR inhibition resulted in reduced phosphorylation of eIF2α, decreased virus titer upon PKR inhibition may also indicate that eIF2α phosphorylation is used as a means to enhance IPNV replication. It was previously shown that translation of some cellular mRNAs is enhanced when eIF2α is phosphorylated. The common features for these mRNAs are: (1) low translation and transcription under normal conditions; (2) possession of translated upstream ORFs (uORFS) at their 5′ region; and (3) at least one of the uORFs is longer than 20 codons and is translated under normal conditions [41]. Ebola virus is able to translate its protein when eIF2α is phosphorylated and the translation of the virus polymerase as well as virus replication was influenced by the presence of uORF. Notably, under high levels of eIF2α phosphorylation, the presence of the uORF enhanced the translation of the L polymerase and removal of the uORF by mutating the start codon resulted in decreased virus replication [42]. IPNV possesses an uORF that encodes for VP5 and overlaps the main ORF in segment A [43]. It is possible that the presence of the uORF will result in enhanced translation under eIF2α phosphorylation. A previous study performed by our group, however, demonstrated that ablation of VP5 expression by mutating the start codon as well as truncation by inserting premature stop codon did not affect virus replication [44]. However, the mutation was made in the second in-frame start codon that was suggested to be the site at which the translation of VP5 is initiated [43]. It remains possible that the first in-frame start codon is the one responsible for the translation control of the main ORF, especially since mutations in the region containing this codon did not yield viable virus [43]. Further studies are therefore warranted to better understand the role of uORF encoding VP5 in the control of virus replication.

The decrease in virus titer was accompanied by a decrease in the number of necrotic cells (or cells with compromised membranes) (Figure 6). One interpretation of these findings could be that the virulent Sp strain of IPNV uses PKR-mediated cellular necrosis to induce cellular lysis and subsequently facilitate virus release. In this case, the anticipation would be that PKR interacts with RIP1 and induce cellular necrosis via the RIP1/RIP3 pathway as previously described [13]. Due to the paucity of reagents and antibodies, we could not further investigate this possibility. However, since the decrease in number of cells with compromised membrane coincided with decreased intracellular virus titers, we hypothesize that a reduced number of cells with compromised membranes is a direct consequence of reduced virus replication (Figure 7), although we cannot completely rule out the former possibility. This latter interpretation is supported by the fact that birnaviruses were shown to possess mechanisms that enable them to perforate cell membranes [45].

Although the induction of eIF2α phosphorylation following IPNV infection has been reported previously [36], no efforts were directed at investigating the mechanisms involved. The eIF2a kinases that activated during IPNV infection are PKR and PERK [35,46]. In this study, we did not detect PKR upregulation post IPNV infection. However, treatment with a PKR inhibitor resulted in decreased eIF2α phosphorylation (Figure 4) and, through a competitive approach (transfection with mutated form of PKR), we also showed a significant reduction of IPNV titer in a permissive cell line (Supplementary Figure S1). This is the same trend as seen for 1 and 0.1 MOI with higher variation between parallels for 0.1 MOI and, thus, is only significant for the highest (primary) infection dose. Upregulation of PKR is not necessary for the PKR function and basal levels of activated PKR are sufficient to induce eIF2α phosphorylation and inhibit translation in response to virus infection [47]. Our findings therefore suggest that PKR participates, at least partially, in eIF2α phosphorylation during IPNV infection. This is in agreement with what has been reported for another member of the family *Birnaviridae*, the infectious bursal disease virus (IBDV), where the expression of VP2 in HeLa cells was associated with PKR activation and subsequently eIF2α phosphorylation [48]. It is therefore possible that VP2 of IPNV plays a similar role. However, the interaction between *Birnaviruses* and PKR seems to be complex as expression of VP3 of IBDV resulted in inhibition of PKR activation and subsequently eIF2α phosphorylation [48]. Nevertheless, in contrast to our study, these results were neither obtained using cell line derived from host species as HeLa cells (of human origin), nor from using virus-infected cells. Further studies are therefore needed to understand the interplay between birnaviruses, PKR activation and eIF2α phosphorylation.

The ultimate goal for a virus infection is to use the cell’s machinery to produce new virus progeny. PKR activation leads to attenuation of global protein synthesis through eIF2α phosphorylation and could therefore have a severe impact on production of a new virus progeny [49]. Consequently, it is not surprising that viruses have developed different strategies to prevent PKR activation [50]. Many viruses, on the other hand, are capable of producing their progenies through cap-independent mechanisms and are thus not affected by the translation inhibition induced by PKR/eIF2α [51]. The replication of IPNV despite eIF2α phosphorylation (Figure 4) is consistent with our previous findings [25] and implies that translation of IPNV possesses a cap independent mechanism to translate its own protein. Since IPNV possesses uORF in segment A as indicated above, another possibility is that this uORF inhibits the translation of IPNV proteins and eIF2α phosphorylation will mediate the bypass of this uORF and help to promote the translation of the second ORF encoding the 107-kDa polyprotein. Further studies are required, however, to investigate these possibilities.

PKR was shown to be responsible for NFkB activation in herpes simplex virus infected cells. This activation participates in the control of virus replication as the virus yields in PKR or NFkB deficient cells were 10-fold higher than in PKR and NFkB sufficient cells [52]. For other different viruses, however, NFkB activation can be used in favor of the virus either as a means to inhibit apoptosis and prolong cell survival in order to have more time for replication or to directly enhance viral replication for viruses that possess NFkB binding sites in their promotor [53]. It was shown previously that NFkB is activated in IPNV infected cells [54]. Although involvement of tyrosine kinase pathway was suggested, the mechanisms involved have not been determined. It is known that PKR induced necrosis requires interaction with RIP1 and is negatively regulated by FADD and caspases [13], both components of the apoptotic signaling [55,56]. RIP1 activation can also lead to NFkB activation [57]. However, NFkB signaling is generally considered as an anti-apoptotic response although in some cases it can also be linked to cell death [57]. In the current study, we found an increase in the number of necrotic cells following IPNV infection while the apoptotic cell number remained unchanged (Figure 6). The mechanism by which cell death is induced during IPNV infection remains obscure and further studies are required to investigate the possible involvement of PKR and NFkB signaling pathways.

The oxindole/imidazole derivative C16 is widely used as specific PKR inhibitor *in vitro*. Recently, it was shown that systemic injection of rats with this inhibitor resulted in *in vivo* PKR inhibition suggesting that it can be used to reverse the undesirable effect of PKR activation in certain diseases such as the neurodegenerative disorder [58]. The activity of this inhibitor against fish PKR has not been previously investigated. The finding that treatment with this inhibitor reduced IPNV titers *in vitro* is interesting and demonstrates that C16 can be used to inhibit IPNV replication as a novel anti IPNV drug target. A similar *in vivo* effect and further demonstration of specificity in addition to safety must, however, first be demonstrated and considered.

## 5. Conclusions

The data presented in this study provides new insights into the interaction between IPNV and PKR. We show that PKR activation may be beneficial to the survival of IPNV since chemical inhibition of PKR using the PKR inhibitor C16 resulted in reduced virus replication. We suggest that C16 interfere with intracellular virus replication events occurring prior to the release stage. Nevertheless, the detailed interaction between PKR and the virus requires further investigation.

## Figures and Tables

**Table 1:** Primers used for PCR.

Name	Primer Sequence	Use	Genbank Accession No.
B actin-F	CCAGTCCTGCTCACTGAGGC	qPCR	AF012125
B actin-R	GGTCTCAAACATGATCTGGGTCA		
IPNV-F	CAACAGGGTTCGACAAACCATAC	qPCR	
IPNV-R	TTGACGATGTCGGCGTTTC		
PKR-F1	TCAACGCATTCTACTGCAC	pGEMT cloning	EF523422.1
PKR-R1	GAAACCCAGCCTAAAACCC		
pET32c-PKR-F	GCGGAATTCGAGATTCCACAAATT	pET32c cloning	
pET32c-PKR-R	GCGAAGCTTTTAGATTGTTCTGTTG		
PKR-F2	ATGAACACAGCCAGAAGAAC	qPCR	EF523422.1
PKR-R2	TTCTCACCTACCACATACCTAC		

## Supplementary Materials

The following is available online at www.mdpi.com/1999-4915/8/6/173/s1, Figure S1: mutcarpPKR effect on IPNV titer in EPC cells.

## Abbreviations

The following abbreviations are used in this manuscript:

IPNV: infectious pancreatic necrosis virus
PKR: phosphokinase R
eIF2a: eukaryotic initiation factor 2 alpha
