# Supplementary material for: PKR Activation Favors Infectious Pancreatic Necrosis Virus Replication in Infected Cells
Source: Viruses. 2016 Jun 21;8(6):173. doi: 10.3390/v8060173 (PMC4926193; doi:10.3390/v8060173)
Supplement: Supplementary file 1 [file viruses-08-00173-s001.pdf]

Supplementary Materials: PKR Activation Favors Infectious Pancreatic Necrosis Virus Replication in Infected Cells

Amr A.A. Gamil, Cheng Xu, Stephen Mutoloki and Øystein Evensen

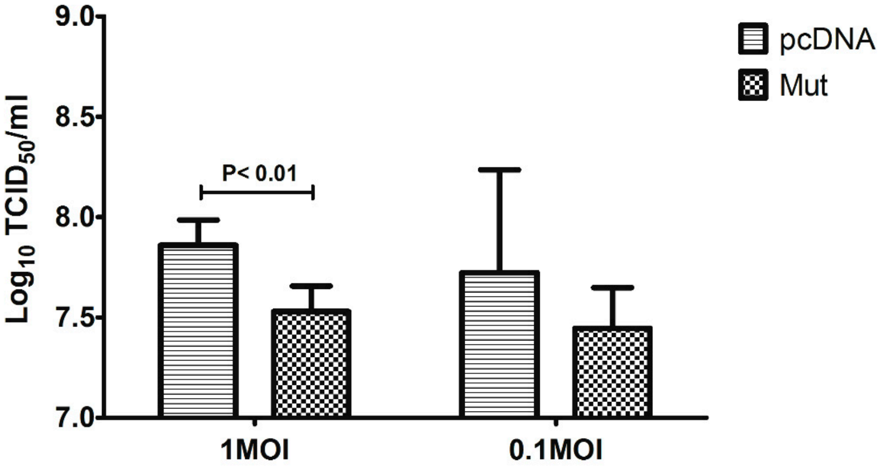

Figure S1. mutcarpPKR effect on IPNV titer in EPC cells. EPC cells in each well of 6-well plates were transfected with a total amount of 2 μg of pcDNA3.1-mutPKR or pcDNA3.1-myc-His plasmids. At 2 h post transfection, the cells were infected with 1 and 0.1 MOI IPNV. Cell supernatants were collected at 72 hpi for virus titration (in CHSE cells; mean + SEM, $n = 3$).

© 2016 by the authors; licensee MDPI, Basel, Switzerland. This article is an open access article distributed under the terms and conditions of the Creative Commons by Attribution (CC-BY) license (http://creativecommons.org/licenses/by/4.0/).
